# Protecting affiliation during pandemic crises: a capabilities approach for public health ethical deliberation

**DOI:** 10.1080/11287462.2026.2668171

**Published:** 2026-05-07

**Authors:** García-Calderó Gemma, Seaward Helene, Elger Bernice Simone, De Clercq Eva

**Affiliations:** a Institute for Biomedical Ethics (IBMB), University of Basel, Basel, Switzerland

**Keywords:** Public health, pandemic, ethics, capabilities approach, social relationships, loneliness

## Abstract

During the COVID-19 pandemic, non-pharmaceutical interventions that affected people's ability to access and maintain social relationships, which philosopher Martha Nussbaum calls “affiliation”, were implemented. However, the WHO has reported that no measures to actively protect social relationships have been put in place. Furthermore, existing pandemic ethical frameworks do not explicitly specify affiliation protection as a relevant ethical consideration. Subsequently, the main aim of this work is to justify why pandemic public health ethical deliberation should aim at protecting people's capability of affiliation, and to present Nussbaum's “capabilities approach” as a suitable theory to support this endeavour. First, we discuss the contributions of the capabilities approach to the ethics of public health, specifically in the context of pandemics. Second, we argue how affiliation informs the core public health ethical principles of respect for individual freedom and equity. This paper provides an original normative account, as (1) it recognises the irreplaceable role of personal relationships, by contrast to other accounts that favour primarily online or non-human interactions, and (2) it builds a conceptual understanding of their role in public health ethics, paving the way for the integration of health and affiliation in future pandemic deliberation.

## Introduction

Non-pharmaceutical interventions are public health strategies that aim at preventing the spread of infectious diseases when no vaccine or treatment is available (The Royal Society, 2023). They are based on two main mechanisms: making social contacts safer—e.g. use of masks, disinfection, ventilation, surveillance—and/or curtailing social contact through isolation of cases or quarantine of contacts (Rehfuess et al., 2023). Non-pharmaceutical interventions have been implemented in different disease outbreaks throughout history, especially quarantine and isolation (Snowden, 2019; Tognotti, 2013). However, the intensity and duration of social contact restrictions during the COVID-19 pandemic of 2020 were unprecedented (Miller & Moss, 2023). These measures were not limited to isolating or quarantining individuals or communities who were infected or exposed to infection but also included those with no infection exposure, embracing society as a whole, with prohibitions on gatherings and the closure of schools and workplaces (Schady et al., 2023). In some countries, strict national “lockdowns” (restrictions on leaving one's home) were implemented for months. Other countries (Mathieu et al., 2020), even faced subsequent re-implementations of lockdowns months after the COVID-19 pandemic was declared by the World Health Organisation (WHO), such as the United Kingdom or Germany (Mathieu et al., 2020). Despite the implementation of non-pharmaceutical interventions being underpinned by the aim of avoiding deaths and preventing overload of the healthcare system (Woolhouse, 2022), these interventions have had an impact on the relational needs of individuals, increasing overall levels of loneliness (Ernst et al., 2022). Some groups were particularly affected; for instance, young people reported a greater increase in loneliness than before the pandemic (Groarke et al., 2020; Wickens et al., 2021).

Loneliness entails a particular deficiency in social relationships (Sønderby and Wagoner, 2013), which have both constitutive and instrumental value (Gheaus, 2022). They are considered a significant component of a “good” human life, which means enjoying a flourishing life judged as fulfilling (Nussbaum, 2001). Furthermore, the goods derived from them, such as attention, affection, and encouragement, are key determinants of psychological and emotional stability and of the development of autonomy (Brownlee, 2020; Gheaus, 2022). Thus, it is noteworthy that the impact on social relationships was widely ignored in the management of the COVID-19 pandemic. This is attested to by the WHO's recent report on national responses to collateral harm caused by the implementation of non-pharmaceutical interventions, which did not include any measures for their protection (World Health Organization, 2024). In this paper, we will refer to the human relational dimension as *affiliation*, a term employed by American philosopher Martha Nussbaum in her “capabilities approach” to describe the ability to live with and toward others and to engage in various forms of social interaction (Nussbaum, 2011, p. 34). Hence, affiliation refers to the possibility of accessing and participating in personal and social relationships. Although Nussbaum also describes a second component of affiliation, which involves non-discrimination and being treated as a dignified living being, we will focus only on the first component.

Even though several ethical frameworks and guidelines to inform decision-making during pandemics have been developed within the discipline of public health ethics following the publication of Influenza pandemic preparedness plans, these frameworks do not explicitly specify affiliation protection as a relevant ethical consideration (Kass, 2005; Kinlaw et al., 2009; Thompson et al., 2006; Upshur et al., 2007). Moreover, they have not been revised since the COVID-19 pandemic. Therefore, there is a conceptual gap regarding the relevance of affiliation for pandemic ethical considerations and principles. These include respect for individual freedom and equitable distribution of burdens, primarily because public health measures are commonly enforced and have inequitable impacts on specific populations (Faden et al., 2025; Gostin & Wiley, 2018). If pandemic ethical frameworks are to be applied in a future pandemic integrating the new insights acquired during the COVID-19 pandemic response, affiliation should be acknowledged as a prominent value to be considered and protected. We believe that showing how affiliation is relevant to the core ethical considerations that inform these pandemic ethical frameworks will help advance a model of public health deliberation that seeks to promote health and protect social relationships without leading to a stark dichotomy among these values. We believe that such an approach is aligned with what Bruce Jennings (2019) named “relational theorising” in public health. To accomplish this endeavour, a normative underpinning suited for public health policy that recognises both health and affiliation needs to inform ethical considerations in pandemics. In this paper, we put forth Martha Nussbaum's “capabilities approach”, a social justice theory that describes a set of basic capabilities governments should promote.

Even though a few authors have developed ethical arguments for the importance of countering loneliness derived from pandemic public health measures, these arguments are not grounded in core public health ethical considerations and therefore do not directly serve the purpose of further developing an understanding of public health that embraces relational theorising (Jennings, 2019). de Vries (2021) argued that states have to take reasonable measures to protect citizens from loneliness when applying social restriction measures, such as lockdowns, by drawing mainly on the associations of loneliness with adverse health outcomes such as anxiety or depression. In contrast to de Vries, who does not rely entirely on human interaction by proposing several ways to make non-human companionship and non-social solutions more widely available (e.g. pets, social robots, and mindfulness workshops), our paper holds that human relationships are irreplaceable. Alternatively, Lederman proposed including loneliness in pandemic health plans (Lederman, 2023a) and acknowledged the unique character of human touch (Lederman, 2023b). Even though we agree with Lederman's proposals, we believe there is a need to provide a deeper justification for how the protection of access and the maintenance of social relationships align with the mission of public health and its ethical principles.

This paper responds to the knowledge gap regarding the role of affiliation in pandemic public health deliberation. Accordingly, the main aim of this work is to explain why pandemic public health ethical deliberation should aim at protecting people's capability of affiliation, by showing its relevance to two prominent public health ethical considerations: respect for individual freedom and equity, through Martha Nussbaum's “capabilities approach” (2011). In line with existing work (Sullivan et al., 2022), this article also intends to provide support for Nussbaum's capabilities approach as an apt theory for integrating affiliation into public health ethical deliberation.

## A capabilities approach for pandemic public health ethical deliberation

### Capabilities approach in public health ethics

In the last two decades, the capabilities approach has inspired works at the core of, or related to, the ethics of public health, especially regarding an understanding of justice (Powers et al., 2006; Prah Ruger, 2009; Venkatapuram, 2013; Wolff & De-Shalit, 2007). Although the conceptual foundations of the capabilities approach were first developed by Amartya Sen (1999), our article focuses on Martha Nussbaum's later contributions to the approach, as set out in her account of affiliation.

Nussbaum's understanding of “capability” transcends the person's inner ability to do and to be, as capabilities refer to “the freedoms or opportunities created by a combination of personal abilities and the political, social, and economic environment” (Nussbaum, 2011, p. 20). As such, a capability is a fundamental freedom that allows one to enjoy a specific function, that is, “the active realisation of one or more capabilities” (Nussbaum, 2011, p. 24). We can think of a particular example of health: a person with a life-threatening disease may have the capability of health by being able to undergo novel treatment, which offers a guarantee of effectiveness. However, this person may decide to refuse the treatment after considering the burdens it would impose as disproportionate, and he or she may eventually decide not to enjoy an improved health status (i.e. not have the corresponding functioning). By granting access to treatment, the government has promoted the capability to pursue a better health status of the person who suffers from that disease, but he or she has ultimately chosen not to receive it. According to Nussbaum, public policies should promote capabilities, enabling citizens to transform them into functioning.

Jennifer Prah Ruger (2009) highlights the benefits of ethical reasoning in public health based on capabilities compared to other ethical approaches. Firstly, the capabilities approach considers human heterogeneity and the fact that persons have different resource needs to attain the same degree of capability. In contrast, the well-known Rawlsian approach, which focuses mainly on resources, does not acknowledge the obstacles to capability and functioning that may be present in people's lives. Secondly, the capabilities approach sets the capability to achieve certain valuable functioning as the focal variable for evaluation. This would be a better indicator for well-being and thus a more reliable guide for priority setting in public policy than utility or personal preferences and desires.

We shall highlight Nussbaum's acknowledgement that not all capabilities have the same value and therefore ought not to be protected and promoted by the government. Those that deserve protection and promotion are called “basic capabilities”, which she features in a list of ten capabilities. This list is Nussbaum's answer to the fundamental question of what makes a life worthy of human dignity. She draws on Aristotle's idea that “satisfaction achieved without choice is unworthy of the dignity of human beings” (Nussbaum, 2011, p. 125). The basic capabilities list is inspired by a series of functionings without which a human life “would be too lacking, too impoverished (…), it could not be a good human life” (Nussbaum, 1992, p. 220). With it, Nussbaum builds a ground-floor conception of the good, which she claims is not metaphysical but derives from self-interpretations and self-evaluations of human beings throughout history and from a wide variety of self-understandings of people across many times and places. Nussbaum is influenced by Aristotelian philosophy, which holds that developing human inner powers to its flourishment is part of the end of human life. Amongst the list of “basic capabilities”, Nussbaum includes physical health and affiliation.

These two capabilities are also considered by Powers et al. (2006) as two central dimensions of well-being (even though they use the term “attachment” instead of “affiliation”, which bears an equivalent meaning, i.e. the formation of bonds of friendship and love, as well as a sense of solidarity with other members of the community). As such, these authors consider promoting health and affiliation through public policy to be a matter of social justice, as these dimensions are essentially important to everyone regardless of their specific life plans and preferences. According to Powers et al. (2006), these two basic dimensions of well-being cannot be separated from public health aims. Therefore, they believe that justice in public health policy requires addressing well-being across its different dimensions. They specifically criticise how traditional theories of justice that inform public health practice usually overlook the fact that human lives are characterised by dependency and interdependency. Therefore, they propose that public policies create the appropriate setting in which people can cultivate bonds of attachment and capacities for fellow feeling with others as a requirement for social justice.

Despite the valuable contributions the capabilities approach has made to public health ethics, only a few authors have advocated its application in the context of a pandemic. Sridhar Venkatapuram (2020) upholds the advantages of considering health as a capability that depends on both individual physical functioning and the external social environment, which varies across heterogeneous individuals and their social conditions. Instead of focusing exclusively on individual safety measures (vaccines, masks, soap), a capabilities approach would shift attention to persons and their capabilities and vulnerabilities in preventing infection in themselves and in others. Therefore, it would promote the implementation of structural changes in the social environment to protect and maintain the capabilities of people to stay healthy. Alternatively, Sullivan et al. (2022) have proposed it as a framework that supports health equity and agency in clinical decision-making of people with intellectual and developmental disabilities and as an ethical model that integrates individual well-being and the common good. Notwithstanding, a closer look at the relevance of the capability of affiliation in this context remains lacking.

Drawing on the existing literature, our paper aims to advance the field by highlighting the relational needs of individuals during pandemics. Specifically, we will focus on two key ethical considerations that commonly underpin public health practice, and in particular, pandemic response: individual freedom and equity (Faden et al., 2025; Marckmann et al., 2015). Through these two prisms, we will argue for the relevance of affiliation in public health ethical deliberation.

### Capability of affiliation in pandemics and freedom

Nussbaum notes that once important functionings have been defined, the question of whether social and political institutions are rendering citizens capable of functioning in these human ways follows. As mentioned above, Nussbaum's capabilities approach stresses that not all freedoms should be given the same weight. The possibility of enjoying a healthy status (which is not the same as being healthy) is a fundamental freedom, a basic capability that, according to Nussbaum, should be protected by society as a condition for social justice. Hence, aligned with public health ethics principles, curtailing other freedoms would be justified, provided that there are no other, less restrictive means to achieve the same effect (Gostin & Wiley, 2016; Upshur, 2002). An infectious disease outbreak poses a risk to people's capability of health, which requires intervention at the population level in order to offer protection to health. These interventions can thwart the exercise of different individual freedoms, imposing some behavioural constraints—e.g. prohibitions on travel or wearing a protective mask in public spaces. However, during the COVID-19 pandemic, these interventions were applied in some contexts, leaving some individuals socially isolated for several weeks and even months (Buecker & Horstmann, 2021; Su et al., 2023). In these cases, the promotion of the capability of health enters into conflict with the protection of the capability of affiliation.

Furthermore, as research on solidarity during COVID-19 attests, some citizens broke the rules to assist others in need of social or psychological support (Hangel et al., 2022; Kieslich et al., 2023). Wollf and De Shalit uphold that “being able to care for others is part of being a person” and thus, “one should have the chance to express oneself, and one's connections with, and care for, others, through appropriate means” (Wolff & De-Shalit, 2007, p. 46). Therefore, situations in which people find themselves in the position of trespassing the law to maintain a minimum level of affiliation indicate a poor policy design that overlooks people's basic capabilities to attain essential functionings. As Wolff and De-Shalit (2007) further argue, opportunities for a particular functioning (for instance, affiliation) are genuine if the person does not have to sacrifice the security of other important functionings to attain it. They therefore maintain that governments should ensure genuine opportunities for essential functionings.

Just as health is a basic capability that calls for promotion and protection, so is affiliation. The capability to maintain personal and social bonds with others is not just any type of freedom, but a fundamental freedom. It is a basic capability key for well-being and flourishing, and a central component of a decent life. In Powers' et al. (2006) conceptualisation of well-being, health and attachment are irreducible dimensions, each of which is a relevant independent moral concern for public health. In line with this view, Nussbaum's capabilities approach stresses the incommensurability of basic capabilities, i.e. the fact that the lack of one cannot be compensated for by an excess of another. Indeed, Martha Nussbaum envisions any situation in which two or more basic capabilities compete as tragic, because any chosen option involves doing wrong to someone (Nussbaum, 2011, p. 37). Pandemic public health policies promoting the capability of health in ways that render individuals unable to effectively engage in social interactions fail to acknowledge basic capabilities' irreducibility. Consequently, in order to guarantee respect for all capabilities, governments should strive to attain a certain threshold for each of them: “when thresholds are set at a reasonable level”, we can “work toward a future in which the claims of all capabilities can be fulfilled” (Nussbaum, 2011, p. 38). Hence, Nussbaum's argument has a clear implication for public health measures applied during pandemics: health should be pursued as an intrinsically valuable capability without overriding people's capability for affiliation, at least to a minimally decent level. We shall address the indeterminacy of this statement later on.

### Capability of affiliation in pandemics and equity

Nussbaum notes that “people have differing needs for resources if they are to attain a similar level of functioning, and they also have different abilities to convert resources into functionings” (Nussbaum, 2011, p. 57). In this regard, she draws a helpful distinction between “internal capabilities”, which are trained and developed traits and abilities, and “combined capabilities”, which constitute the internal capabilities plus the social, political, and economic conditions that enable the transformation of capabilities into functionings. Thus, we can view non-pharmaceutical interventions as promoters of the combined capability of health, as they shape social conditions to reduce the risk of acquiring the infectious disease, thereby enabling individuals to maintain a healthy state. However, as mentioned above, its implementation can affect other capabilities. In the case of affiliation, this effect can be greater for specific individuals if we consider the state of their internal capability of affiliation.

As asserted by socio-emotional selectivity theory and the theory of emerging adulthood, adolescents and young adults undergo a maturation process that involves exploring and understanding the world, which requires interactions with peers from broader social circles (Arnett, 2000; Carstensen, 1995). In addition, decreased contact with friends is associated with higher levels of loneliness in this group than in other age groups (Nicolaisen & Thorsen, 2017). As such, we can consider that young individuals are generally in a life phase of developing their social skills and network, building up their internal capability of affiliation, and thus have a special need to establish social relationships. Subsequently, neglecting these needs when implementing public health measures can prove especially harmful to them. Nussbaum recognises affiliation, together with practical reasoning, as capabilities whose deprivation brings about “corrosive disadvantage”, namely, particularly “large effects in other areas of life” (Nussbaum, 2011, p. 44). Evidence from the COVID-19 pandemic supports this statement by showing an association between perceived social isolation and adverse mental health outcomes (Mann et al., 2022; Park et al., 2020), especially among the young population (Dunn & Sicouri, 2022; Loades et al., 2020). Therefore, considering affiliation when developing public health measures is of utmost importance to prevent disadvantages for specific population groups, such as the young population, which is at the heart of the moral endeavour of public health practice (Powers et al., 2006).

Furthermore, the need for cultivating personal relationships may become more prominent in certain moments in life where one experiences some added vulnerabilities —e.g. physical, psychological, relational, and existential (Sanchini et al., 2022), such as at the end of life, during the process of bereavement, or when oneself or a loved one is confronted with illness. People who grieved during the COVID-19 pandemic in the United Kingdom, for instance, have reported high needs for help with loneliness and social isolation, as well as for comfort and reassurance (Harrop & Selman, 2022). Parents of children with cancer described their isolation, both from their spouses and children, as only one visitor at a time was permitted to the ill child's room and from their community, as they could not rely on it for support (Sutherland-Foggio et al., 2024). In addition, non-pharmaceutical interventions restricting social contacts prevented many people who were dying from being accompanied by their loved ones (Parks & Howard, 2021). Dying in isolation implies thwarting the capability of the affiliation of people who might most desperately need it, which, from the perspective of the capabilities approach, is a violation of dignity. Protecting the capability of affiliation to a certain threshold in these situations would be, in our view, one of those instances in which “the fundamental value and dignity of each human being may demand the implementation of public health policies that are neither optimally efficient nor cost-beneficial”, as stated in the “Principles of the Ethical Practice of Public Health” adopted by the American Public Health Association, (2002) (p.3).

## Discussion

The need for a strong public health strategy to combat a pandemic is not called into question. The question that emerges is, rather, what lessons we should learn from the COVID-19 pandemic concerning the aspects to be considered when devising non-pharmaceutical interventions to avoid, in the future, as far as possible, the harmful effects on individuals' relational needs. This is of utmost importance considering the probability of facing a new pandemic, which might require the implementation of non-pharmaceutical interventions for a long time before a vaccine or treatment will be available (Coccia, 2023; Iserson, 2020). As discussed in the introduction, existing pandemic ethical frameworks and guidelines, which have not been amended since COVID-19, do not explicitly recognise affiliation protection as a relevant ethical consideration. In this paper, we argue that ethical deliberation during pandemics should explicitly acknowledge the impact of pandemic measures on people's ability to form and maintain social relationships. Therefore, we believe that pandemic public health strategies should protect this ability to a certain extent. In what follows, we offer a brief exploration of how our analysis could contribute to the improved implementation of some existing pandemic ethical frameworks (Kass, 2005; Kinlaw et al., 2009; Thompson et al., 2006). By doing so, we aim to show how considering affiliation through the lens of the capabilities approach offers a more thorough ethical evaluation.

The public health ethical framework applied to pandemic preparedness plans by Nancy Kass (2005) provides a structure to think through a series of ethical issues derived from such a public health challenge. The framework is divided into six steps, which ask questions regarding (1) goals, (2) effectiveness of interventions, (3) potential burdens, (4) burden minimisation, (5) fair implementation and just distribution, and (6) fair procedures for creating plans. As examples of the goals of pandemic public health measures, Kass proposed reducing morbidity and mortality and maintaining society's infrastructure to the extent possible. The fact that protecting affiliation is not considered one of the framework's main goals may explain the limited attention to social needs across its different sections, which only briefly note that liberty restrictions may pose a psychological burden and that the needs of those who are isolated must be anticipated. Integrating the protection of the capability of affiliation into the framework from the very first step of goal setting would raise additional questions for the rest of the sections, orienting action towards securing safe means of personal and social interaction, especially for those who are most in need.

Kinlaw et al. (2009) include in their ethical guidelines general principles similar to those proposed by Kass (2005). However, they offer further ethical considerations for the development of interventions that curtail individual freedom and create social distancing. The focus is on describing grounds for limiting personal freedom and preventing undue governmental interference. Overlooking the fact that affiliation is a basic capability that should be promoted means missing out on potential steps the government could take to guarantee individuals' well-being.

Including the protection of the capability of affiliation as an ethical goal sheds light on another aspect of the value of individual freedom. It goes beyond being considered a value to be protected from external interference. Public health ethical frameworks usually conceptualise autonomy as a single, broad concept that should be weighed against the pursuit of collective benefit. This approach does not distinguish between the different goods towards which freedom can be oriented (Marckmann et al., 2015)—e.g. affiliation with others, travelling, and enjoying recreational activities. However, the capability approach acknowledges that when this human power is directed towards central functionings (i.e. basic capabilities), such as affiliation, public policies should strive to promote it. This may require positive intervention. Thus, even if promoting the capability of freedom might call for the restriction of other freedoms, ethical evaluation is not limited to its proportionality but should acknowledge and promote all of the irreducible dimensions of well-being—or basic capabilities—as discussed by public health authors inspired by the capabilities approach (Powers et al., 2006; Wolff & De-Shalit, 2007).

Finally, the ethical framework by Thompson et al., (2006) is divided into two lists: one focusing on the ethical decision-making process and the other on ethical values. The list of ethical values includes a description of equity and individual liberty but does not explicitly mention the potential impact on social needs of pandemic measures.

This article has demonstrated the relevance of the capability of affiliation to the principles of respect for individual freedom and equity. Therefore, existing pandemic ethical frameworks should include, within the specification of these ethical principles, a requirement to assess the ability of people to maintain a minimum level of social relationships and the particular needs of groups more vulnerable to loneliness.

The openness in defining capabilities is an intended aspect of Nussbaum's framework, as she considers this to be precisely the role of deliberation, which each country should take on so as to specify capabilities in its own context (Nussbaum, 2011, p. 74). However, the fact that basic capabilities are defined in broad terms and that there is no clarification of the meaning of an appropriate threshold to be respected makes it difficult to describe specifically what these courses of action would look like. Sullivan et al. (2022) uphold that the capabilities approach supports integrating “interdisciplinary inputs into planning responses to pandemics that seek the necessary balance between addressing a public-health threat and promoting overall human well-being” (Sullivan et al., 2022, p. 121). Indeed, inputs from the social sciences and humanities can have inestimable value in shaping recommendations that acknowledge affiliation and, therefore, are an essential piece of public health deliberation rooted in the capabilities approach.

Specific practical recommendations are beyond the scope of this paper, as this would require integrating interdisciplinary empirical evidence from both epidemiology and social sciences. However, we would like to mention some suggestions that have been proposed as possible examples of concrete ways to apply the capabilities approach.

The epidemiologist and UK government advisor during COVID-19, Mark Woolhouse, has proposed to focus first on making social contact safer rather than curtailing it. This could be attained by improving the isolation of cases and quarantining of their contacts, and when necessary, applying increasingly restrictive social distancing measures with special caution regarding their strictness and duration (Woolhouse, 2022). Concerning vulnerable populations such as frail and infirm people, he notes that the isolation of social contacts is an inappropriate measure for these groups, who require care. Inspired by the concept of “precision public health”, which involves providing the right intervention, at the right time, to the right people, Woolhouse proposes a “cocooning” strategy. This approach would involve providing special health protection for their carers, thereby indirectly enhancing health protection and some degree of affiliation amongst these groups (Woolhouse, 2022). We suggest that the concept of “precision public health” may also be applied to the young population, maximising the availability and use of open and other safe spaces that enable social interaction while posing minimal infection risk.

Long et al. (2022) emphasise that *physical* distancing need not imply social distancing, as several forms of social proximity entail minimal risk and are key for cultivating relationships, such as conversations while walking outdoors. In addition, measures with smaller relational costs, such as restrictions on international travel, should be prioritised (Long et al., 2022). Finally, Lederman (2024) claimed that special attention should be given to people who are about to die, enabling those who do not desire to die alone or long enough to have particular persons next to them to accomplish those wishes. He explicitly recognises that allowing family members to access the deathbed can involve enabling human touch; notwithstanding, he upholds that drawing on empirical data and anecdotal evidence, “creative solutions may be sought that allow human touch while not necessarily increasing public health risk significantly” (Lederman, 2024, p. 290).

We would like to acknowledge a notable limitation of our presentation of Nussbaum's capabilities approach, which is the lack of acknowledgement of the importance of people's willingness to respect others' capability of health. Indeed, the fact that Martha Nussbaum assigns moral value to people's ability to freely develop their capabilities calls for solidarity within society (Kieslich et al., 2023). This means that people should have a common understanding of the importance of following certain behaviours that enable others to protect their capability of health. Therefore, we believe that pandemic public health deliberation aimed at protecting affiliation should go hand in hand with deliberation to promote solidarity. We consider this a topic for further research that warrants attention.

## Conclusion

Non-pharmaceutical interventions implemented during the COVID-19 pandemic had a strong impact on people's needs for social relationships, particularly among certain groups, such as young people and those facing situations that require special relational support. Following Martha Nussbaum's “capabilities approach”, this paper has shown that protecting the capability of affiliation (the possibility of having access to and participating in personal and social relationships) during pandemics is a moral endeavour that responds to the public health ethical principles of respect for individual freedom and equity in the distribution of burdens. Thus, as a relevant aspect of public health ethics, it should be considered during deliberation without implying a trade-off with the capability of health. The deliberative process should rather identify courses of action that promote both capabilities to a certain threshold, offering stronger support to those groups with greater needs. Further specification of the appropriate threshold and possible courses of action is part of the deliberation process that should be conducted at the national level, integrating scientific information with a sensitive assessment of contextual needs.
